# Divergence-Based Segmentation Algorithm for Heavy-Tailed Acoustic Signals with Time-Varying Characteristics

**DOI:** 10.3390/s21248487

**Published:** 2021-12-20

**Authors:** Aleksandra Grzesiek, Karolina Gąsior, Agnieszka Wyłomańska, Radosław Zimroz

**Affiliations:** 1 Faculty of Pure and Applied Mathematics, Hugo Steinhaus Center, Wroclaw University of Science and Technology, Wyspianskiego 27, 50-370 Wroclaw, Poland; 242966@student.pwr.edu.pl (K.G.); agnieszka.wylomanska@pwr.edu.pl (A.W.); 2 Faculty of Geoengineering, Mining and Geology, Wroclaw University of Science and Technology, Wyspianskiego 27, 50-370 Wroclaw, Poland; radoslaw.zimroz@pwr.edu.pl

**Keywords:** time-varying characteristics, heavy-tailed distribution, segmentation, acoustic signal, grinding

## Abstract

Many real-world systems change their parameters during the operation. Thus, before the analysis of the data, there is a need to divide the raw signal into parts that can be considered as homogeneous segments. In this paper, we propose a segmentation procedure that can be applied for the signal with time-varying characteristics. Moreover, we assume that the examined signal exhibits impulsive behavior, thus it corresponds to the so-called heavy-tailed class of distributions. Due to the specific behavior of the data, classical algorithms known from the literature cannot be used directly in the segmentation procedure. In the considered case, the transition between parts corresponding to homogeneous segments is smooth and non-linear. This causes that the segmentation algorithm is more complex than in the classical case. We propose to apply the divergence measures that are based on the distance between the probability density functions for the two examined distributions. The novel segmentation algorithm is applied to real acoustic signals acquired during coffee grinding. Justification of the methodology has been performed experimentally and using Monte-Carlo simulations for data from the model with heavy-tailed distribution (here the stable distribution) with time-varying parameters. Although the methodology is demonstrated for a specific case, it can be extended to any process with time-changing characteristics.

## 1. Introduction

Many real-world systems change their parameters during the operation. It could be a continuous progressing change (like start up of the machine) or switching from regime A to another regime B (for example, loaded/unloaded machine). Analysis of such time-varying processes (using acquired data) is difficult. If the analyzed data have a complex structure, then before further analysis they should be divided into simpler subsignals. That requires the use of different methods. Such approaches are commonly called signal segmentation [1,2,3].

The task of segmentation considered in this paper is more general than the classical segmentation, where one is looking for a moment where characteristics of the signal have changed. Usually basic statistics (mean, variance, kurtosis, etc.) or more advanced features are used as criteria for splitting the signal into two or more homogeneous parts.

As mentioned, the reasons for using segmentation may be very different. It is commonly used as pre-processing for a non-stationary signal which consists of stationary segments. In real data, there are many situations related to this problem. A good example is a machine that may change the regime of operation or a car with a manual gearbox where changing gear may be associated with a change of internal conditions in the system. Thus, it requires separate treatment.

Signal segmentation is very advanced in speech processing where a single word or even a vowel should be extracted and recognized. Detection of the event in the signal (seismic shock [4,5,6,7,8,9,10,11], where the classical algorithm is the STA/LTA [12], mammal sound in underwater [13], speech [14,15], biomedical signals [16], various processes [17]) may also be linked to signal segmentation. One should detect in the signal such a part, where extra information appeared. It is often used later for further processing. Other interesting applications include: physics [18,19,20,21,22], and biology [23,24,25,26,27]. The segmentation problems appear also in condition monitoring [28,29,30,31,32].

Complexity of segmentation may be different, from quite simple statistic based decision making, especially if differences in the data for different subprocesses are clear to notice (as a change of mean or variance) to very advanced techniques for a more complicated process. Interesting approaches used for the problem related to the changing of the location parameter (like mean) can be found in [33,34,35,36,37,38,39,40] while the methods for changing the scale parameter (like variance) are presented, for instance, in [33,41,42,43,44,45,46,47,48]. A specific case is related to heavy-tailed processes [28,29,30,31] where we expect the impulsive behavior of the corresponding data. In that case, the simple statistics are useless. Moreover, the differences between processes are easy to find by visual inspection, but still the algorithmization of segmentation is challenging. Michalak et al. [31] introduced examples of such heavy-tailed process in engineering, where mineral processing machines and vibration processing have been investigated. Other interesting examples are related to aircraft noise segmentation [49] and financial data analysis using non-stationary compound Poisson processes [50].

In this paper, contrary to the classical segmentation problems, we consider a generalized case by assuming that the parameters of the model behave according to certain functions that stabilize at some level with time. Therefore, the change of regime in the data occurs smoothly. Moreover, the mentioned parameters corresponding to the scale and impulsiveness of the data, change not only according to some deterministic function, but we also observe certain random fluctuations in their values. Therefore, we consider the problem of segmenting the independent observations derived from the distribution with randomized parameters.

In the theory of probability and in statistics, there is a class of distributions resulting from the assumption that a random variable is distributed according to a certain parametrized distribution for which some or all parameters are random. Such distributions are called compound probability distributions. If a scale parameter is randomized, the resulting distribution is also called a scale mixture. In the literature, there are examples of well-known probability distributions derived in this way, for instance, the nonstandardized t-Student distribution (Gaussian distribution with inverse gamma distributed variance), the Laplace distribution (Gaussian distribution with exponentially distributed variance), the negative binomial distribution (Poisson distribution with gamma distributed rate parameter), or the beta-binomial distribution (binomial distribution with beta distributed probability of success). Lee et al. [51] provided a review of the recent developments in the field of scale mixture distributions and an overview of certain examples. Distributions of this type are also used in the real-world data analysis [52,53,54,55,56,57].

The systems with randomized coefficients are considered also in the analysis of the stochastic processes. We mention here the autoregressive time series with random coefficients [58,59,60,61,62] and the Poisson process with random intensity called Cox process or doubly stochastic Poisson process [63,64,65]. In the literature, the authors consider also the fractional Brownian motion with a randomized diffusion parameter called the diffusing diffusivity model [66,67,68].

Segmenting the signals with a smooth transition from one process to another seems to be a more complicated task than the classical case of segmentation mentioned earlier. The problem is even more complex when two parameters of the distribution change. This work proposes a segmentation method for such signals which relies on comparing the probability density functions (pdfs) calculated in moving windows. It is based on the assumption that when the distribution of the data stabilizes, the pdfs are similar. It is important to emphasize that this procedure does not split the entire signal only into homogeneous parts due to the nature of the signal. Nevertheless, the method allows indicating homogeneous fragments of time series that correspond to the parts of the signal with stabilized parameters.

In the proposed procedure, we divide the original signal into segments with a priori predefined length. For each segment, we calculate its empirical pdf and utilise the divergence (called the Jeffreys distance) to measure the distance between the calculated empirical densities. Finally, we differentiate the Jeffreys distance and use log-likelihood ratio to detect the change point. The introduced segmentation algorithm is applied to real acoustic signals acquired during coffee grinding. The proposed procedure can be summarized in three steps: the estimation of the pdf for data corresponding to the selected segments, calculation of the divergence measure, and identification of the change points for data representing the empirical divergence using one of the classical segmentation algorithm. Justification of the methodology has been performed experimentally and using Monte-Carlo simulations for data from the model with heavy-tailed distribution (here the stable distribution) with time-varying parameters. Although the methodology is demonstrated for a specific case, we hope it is universal and can be extended to any process with time-changing characteristics. Example of such a signal could be seismic signal (vibrations with damping), speech signal, or vibrations in mechanical systems with processes as cutting, compressing or crushing.

The rest of the paper is organized as follows. In Section 2 we formulate the problem and provide information about the performed experiment. In Section 3 we present the methodology used in the paper, including the preliminary study, a reminder of the stable distribution and divergences. Moreover, we introduce the steps of the proposed segmentation procedure. Section 4 is devoted to the analysis of the real signals acquired during coffee grinding, while in Section 5 we present the results of the simulation study. In Section 6 we discuss the results. Section 7 concludes the paper.

## 2. Problem Formulation

In this paper, we consider a non-stationary, highly impulsive, and energetic signal with the distribution stabilizing close to Gaussian. To confirm such an assumption, some preliminary analysis of real data has been performed, see Section 3.1.

The transition between the process with property A ( later called process A) and the process with property B (process B) is smooth, as there is no sharp boundary. It makes change recognition difficult. We may say that the process at time *t* is more A than B or vice versa. However, as the transition is nonlinear and at some point the transition stabilize, it may be use as a criterion for segmentation.

As an illustration, we will use the acoustic signals captured during a grinding of coffee beans in a grinder. At the beginning, due to the cutting of beans by sharp spinning knives in the grinder, the process is very noisy and cutting is an impulsive process. Once the coffee beans are ground, the material in the grinder is more powdery than grainy. Thus, the acoustic signature of the grinding process is no more impulsive nor energetic. The key issue is how much time we should perform the grinding to achieve a satisfactory structure of the ground coffee. To validate our procedure, many experiments have been performed with various durations of grinding and after each of them a photo of the structure of coffee was taken and analyzed. A discussion on that is presented in Section 6. Finally, we proposed a model of the real signal and Monte Carlo simulations have been performed, too.

From the mathematical point of view, the problem can be formulated in the following way. Let us imagine that the given observations correspond to some theoretical time series (i.e., a process with discrete time) ${\left\{X_{t}\right\}}_{t\in Z}$ that is defined as follows

(1) $$\begin{array}{c} X_{t}=Z_{t,\theta },\;\;t\in Z, \end{array}$$

where for each t,s∈Z the random variables $Z_{t,\theta }$ and $Z_{s,\theta }$ are independent and have the same distribution. The θ is the parameter of the distribution (in one- or multidimensional space), however we assume that it depends on the time point, i.e., one can write θ=θ(t). The simplest case is when θ is a constant value. In that case, the segmentation is not needed, as the data are homogeneous and constitute a sample of independent identically distributed (i.i.d.) time series. The relatively simple case is also when θ(t) is a constant, one- or multidimensional-valued, function on intervals. In such a case, the segmentation of the corresponding set of observations seems to be relatively easy and reduces to the identification of the points when the parameter θ(t) changes its value. In this case, we can determine the structure change point by analyzing some statistics—depending on the interpretation of the parameter θ, e.g., the estimator of the θ parameter, calculated for the given time windows. Clearly, in the case when the θ(t) parameter is a one-dimensional valued function, the segmentation is simpler than it is for a two- or even multidimensional one. In that case, one considers the problem of structure break point detection in the multidimensional space.

The case when the θ(t) is a constant function on the interval can be generalized to the case when it is any deterministic function (one- or multidimensional valued). In such a case, the segmentation (i.e., division of the set of observations into homogeneous parts) seems to be much more difficult. The segmentation gives us quasi-homogeneous parts, i.e., the parts when the θ(t) parameter has relatively small fluctuations and the behavior of θ(t) is significantly different for the segmented parts. The problem seems to be more complicated when θ(t) is a multidimensional valued function or a random variable. In that case, advanced statistical methods need to be applied to identify the significant change in the signal. The analysis of the estimator of θ(t) for a given time window may not be enough and other statistics should be examined to identify the structure change point. This is the case considered in this paper. As an example, distribution in which the parameter change in time is the stable one, is useful for the analysis of heavy-tailed distributed data. More details of this distribution are presented in the next section. However, this methodology can be extended to any other distribution.

### Experimental Illustration of the Problem

**Figure 1:**
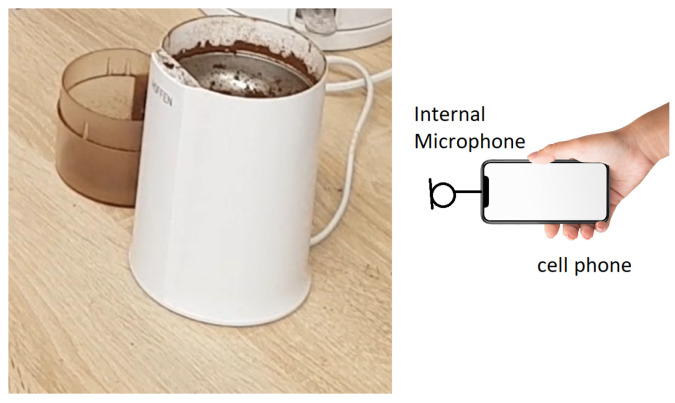
Acoustic signal measurements from coffee bean grinding using a mobile phone.

To illustrate the problem and to validate our procedure using experimental data, we perform several experiments related to coffee bean grinding. Using a popular coffee grinder for domestic use, we prepared dozens of coffee samples with comparable volume and quality parameters. For each sample, the acoustic signal has been registered using a mobile phone, see Figure 1. It was found that approx. 30 s is enough to obtain coffee powder. Conditions of each experiment (not critical here) were approximately similar: the same amount of coffee, the same type of coffee, the same data acquisition device located in the same position and direction, c.a. 1 m from the grinder. During the experiment, one may hear how a “sharp” sound related to “cutting” of coffee beans by rotating knifes in the grinder is changing into a much lower level of noise with a rather narrow-band character (related to rotating knifes). For several samples, grinding was stopped at T = [5, 10, 15, 20, 25, 30] and photos of coffee bean fragmentation phase have been made for validation purposes. After acquisition, the data were transferred to Matlab where appropriate algorithms have been applied. Also all numerical experiments have been performed in Matlab.

## 3. Methodology

The acoustic signals analyzed in the paper, obtained through the experiment described in the previous section, show some special properties. It should be emphasized that the mentioned experiment was conducted many times and the nature of the data is repetitive. For illustration purposes, we selected several realizations, which are subject to preliminary investigation in the next sections. Then, on the basis of the observations made during the initial analysis, a method of data segmentation is proposed.

### 3.1. Preliminary Study

**Figure 2:**
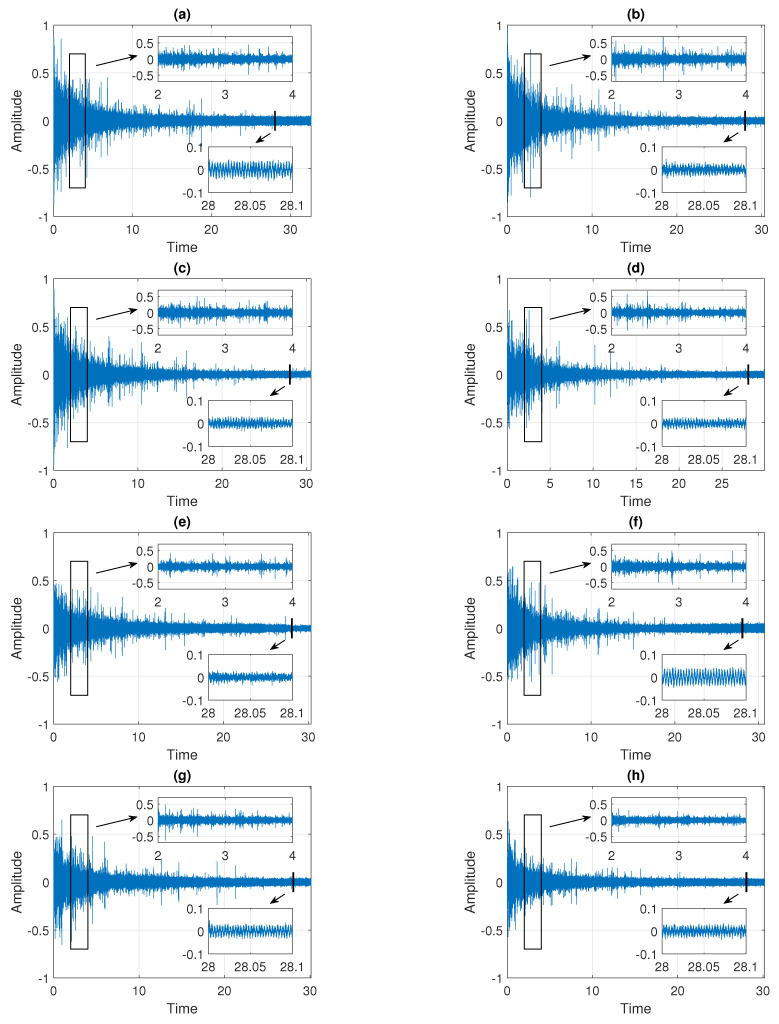
Trajectories of eight raw signals. Panels (**a**–**h**) correspond to Signals 1–8, respectively.

In the paper, we examine eight signals denoted as Signals 1–8 presented in the subsequent panels of Figure 2. The sampling frequency is equal to 44,100 Hz and the length of the trajectories is about 30 s. As it can be seen in Figure 2, the data are clearly non-stationary. The characteristics of the signals change with time, i.e., the amplitude and the observation range decrease over the observed period. From a statistical point of view, we can say that the values of dispersion measures (such as standard deviation, interquartile range, and average deviation from the mean) vary over time, whereas the values of mean and median remain constant. Moreover, the data have an impulsive character, as demonstrated by the outliers present in the signals, see the zoomed fragments of the plots in Figure 2 (between 2 s and 4 s). Nevertheless, the number of impulses and their amplitude also decrease in time. In the zoomed fragments of the plots in Figure 2 (around 28 s), one can observe also some deterministic trends that are present in the data.

Following the above-mentioned remarks, we can conclude that the distribution behind the data presented in the subsequent panels of Figure 2 belongs to the class of heavy-tailed distributions for which large observations (called outliers) are more likely to appear than in the Gaussian case. A classical example of such a distribution is the stable one. Due to the Generalized Central Limit Theorem, it constitutes a natural extension of the Gaussian distribution. Moreover, its tail decays to zero according to a power-law function (slower than exponentially decaying tails in the Gaussian case) and therefore it is often used as a model for data with impulsive behavior. Here, we propose to use this distribution to describe the considered real signals presented in Figure 2. At the same time, we should take into account that, since the properties of the data change in time, the parameters of the distribution are not time-constant. More information about the stable distribution and the proposed model can be found later in this paper, see Section 3.2 and Section 3.3.

The main goal of the paper is to introduce a procedure leading to the segmentation of the signals presented in Figure 2. The proposed method is designed to answer the question of when the signal’s properties stabilize by decomposing the data into segments in which the probability distributions of the signal show similar features. See Section 3.5 for more details.

### 3.2. The Stable Distribution with Changing Parameters

The stable distribution (also called α-stable or Lévy stable), introduced by Lévy and Khinchine in the 20 s and 30 s of the previous century [69,70], is considered as an extension of the Gaussian distribution. It can be defined in four equivalent ways [71,72,73,74,75,76] and one of the definitions concerns Generalized Central Limit Theorem, stating that the stable distribution is the limiting distribution for normalized sums of independent random variables with identical distribution and diverging variance. Here, we present the characteristic function of the stable random variable *Z*, which provides the parameters of the distribution,

(2) $$\begin{array}{c} E\left[\exp\{i\theta Z\}\right]=\left\{\begin{array}{cc} \exp\left\{-\sigma^{\alpha }{\left|\theta \right|}^{\alpha }\left(1-i\beta \mathrm{sign}\left(\theta \right)\tan\left(\frac{\pi \alpha }{2}\right)\right)+i\mu \theta )\right\} & \mathrm{if}\alpha \neq 1,\\ \exp\left\{-\sigma |\theta |(1-i\beta \frac{2}{\pi }\mathrm{sign}\left(\theta \right)\ln|\theta |)+i\mu \theta )\right\} & \mathrm{if}\alpha =1, \end{array}\right. \end{array}$$

where

(3) $$\begin{array}{c} \mathrm{sign}\left(\theta \right)=\left\{\begin{array}{cc} 1 & \mathrm{if}\theta >0,\\ 0 & \mathrm{if}\theta =0,\\ -1 & \mathrm{if}\theta <0. \end{array}\right. \end{array}$$

The parameter α is called the stability index and takes values in (0,2], σ is the scale parameter greater than 0, β is the skewness parameter in [−1,1], and μ is the shift parameter taking values in R. For β=μ=0 the distribution is called symmetric and the characteristic function simplifies to the following one

(4) $$\begin{array}{c} E\left[\exp\{i\theta Z\}\right]=\exp\left\{-\sigma^{\alpha }{\left|\theta \right|}^{\alpha }\right\}. \end{array}$$

One can notice that for α=2 one obtains the Gaussian distribution with mean equal to μ and standard variation equal to $\sqrt{2}\sigma$. However, it is important to emphasize that for the non-Gaussian case, the distribution differs significantly from the Gaussian one [77]. As it was mentioned in the previous section, for α≠2 the distribution tails decay as power-law functions and therefore the random variables take extreme values with greater probability than in the Gaussian case. Moreover, the stability index regulates the rate of tail convergence, therefore, the smaller α is, the more impulses are present in the data. Because of that, the stable distribution can be used to model the signals with impulsive behavior. Moreover, for α≠2 the variance of *Z* diverges and the first moment is finite only for 1<α≤2. Therefore, many classical tools and techniques cannot be applied for the non-Gaussian distribution, e.g., the classical measures of dependence. It is worth noticing that the pdf of the stable distributed random variable exists and is continuous, however, it is not given in an analytical form in general.

In the presented simulation study, we assume that the considered observations, after a certain pre-processing step mentioned at the beginning of Section 4, can be treated as a sequence of independent stable distributed random variables. However, since one can notice that the characteristics of the signals change in time, we cannot assume that the subsequent random variables are equally distributed. Therefore, we assume here that the distribution of the data changes in segments, which is equivalent to the fact that the parameters of the distribution, α, σ, β, and μ, change in those segments.

### 3.3. Signal Parameters Identification and Modelling

To identify how the parameters of the distribution change in time, we propose to fit the stable distribution to the signals in narrow windows of length *L*. Then, the observations within each such segment are assumed to be independent and identically distributed, and the parameters of the distribution may change as the window moves. To estimate α, σ, β and μ in each segment, we propose to use the regression-type method introduced in [78], see the results presented in Section 4. Based on the outcomes obtained for the real signals, in Section 5 we propose a model of the signal, which is used to perform the simulation study. For the simulated signal, we assume that the parameters of the stable distribution change not only in a deterministic way but the values are also disturbed by some random noise. Moreover, we assume that at a certain moment the parameters of the distribution remain unchanged. More information about the simulated signal, including its generation and the performance of the segmentation method, are provided in Section 5.

### 3.4. Divergence Measure

In this section, we present the statistics on which the proposed segmentation procedure relies. In probability theory, the similarity between two probability distributions can be quantified by means of divergences that measure the distance between the pdfs. However, the concept of divergence (called also contrast function) is not as strong as the notion of distance. The divergences do not have to be symmetric in the arguments nor satisfy the triangle inequality. An essential class of contrast functions are the *f*-divergences defined as follows [79,80,81]

(5) $$I_{f}\left(p\left(x\right),g\left(x\right)\right)=\int_{R}g\left(x\right)f\left(\frac{p(x)}{g(x)}\right)dx.$$

The functions p(x) and g(x) in Equation (5) are the pdfs corresponding to the two probability distributions under consideration and f(t) is a continuous convex real function on $R_{+}$ such that f(1)=0. The divergences defined in this form are always non-negative. Moreover, the function given in Equation (5) is equal to zero if and only if the pdfs p(x) and g(x) coincide (take the same values for all arguments), which corresponds to the case when the probability distributions are the same. More properties of *f*-divergences one can find, for example, in [81,82].

In this paper, to evaluate how the probability distribution of data changes over time, we use one specific measure, which belongs to the class of *f*-divergences defined above, called the Jeffreys distance and is defined in the following way [83]

(6) $$J\left(p\left(x\right),g\left(x\right)\right)=\int_{R}{\left(\sqrt{p(x)}-\sqrt{g(x)}\right)}^{2}dx,$$

which corresponds to $f\left(t\right)={\left(\sqrt{t}-1\right)}^{2}$ in Equation (5). We mention here that some authors refer to the divergence defined in Equation (6) as the Hellinger distance. One can notice that the considered statistics is symmetric in the arguments, i.e., J(p(x),g(x))=J(g(x),p(x)), and takes values between 0 and 2 with the minimum value corresponding to the case when p(x)=g(x) for each x∈R, and the maximum value when p(x) is equal to zero for every *x* for which g(x) is nonzero and vice versa. In practice, for the empirical data the pdfs in Equation (6) are replaced by their estimators denoted by $\hat{p}\left(x\right)$ and $\hat{g}\left(x\right)$. Therefore, one calculates the empirical counterparts of the Jeffreys distance defined in Equation (6), namely

(7) $$\hat{J}\left(p\left(x\right),g\left(x\right)\right)=\sum_{i=1}^{n}h{\left(\sqrt{\hat{p}\left(x_{i}\right)}-\sqrt{\hat{g}\left(x_{i}\right)}\right)}^{2},$$

where $x_{1},x_{2},\ldots ,x_{n}\in R$ are the arguments of the pdfs and *h* denotes the step, i.e., $h=x_{i}-x_{i-1}$ for i=2,…,n. To estimate the pdf p(x) (or analogously g(x)) in Equation (7) one can use the kernel density estimator of the following form [84,85]

(8) $$\hat{p}\left(x\right)=\frac{1}{Nk}\sum_{j=1}^{N}K\left(\frac{x-Y_{j}}{k}\right),$$

where $Y_{1},Y_{2},\ldots ,Y_{N}$ is the sample from which we estimate the pdf, x∈R, K(·) is the non-negative kernel smoothing function, and *k* is the bandwidth. The kernel smoothing function determines the shape of the curve used to estimate the pdf. We use the Gaussian kernel of the following form

(9) $$K\left(x\right)=\frac{1}{\sqrt{2\pi }}\exp\left\{-\frac{x^{2}}{2}\right\},$$

for x∈R the bandwidth is chosen using the Silverman’s rule of thumb [86]. The kernel density estimator is implemented in most programming languages. We use the function “ksdensity” in Matlab.

### 3.5. Segmentation Procedure

In this section, we describe the proposed segmentation algorithm. The methodology relies on comparing the pdfs using the Jeffreys distance presented in Section 3.4 and segmenting the Jeffreys distance increments by means of the log-likelihood ratio (LLR) method. The subsequent steps of the procedure are as follows:

1. Divide the signal into *M* segments of length equal to *L*.
2. Estimate the pdf in each segment, ${\hat{p}}_{1}\left(x\right),\ldots ,{\hat{p}}_{M}\left(x\right)$.
3. Estimate the pdf corresponding to the last *L* samples in the signal, ${\hat{p}}_{*}\left(x\right)$.
4. Calculate the Jeffreys distance between the pdfs in subsequent segments and the pdf of the last *L* samples,
  (10) $$\left(\hat{J}\left({\hat{p}}_{1}\left(x\right),{\hat{p}}_{*}\left(x\right)\right),\;\;\ldots \;\;,\;\;\hat{J}\left({\hat{p}}_{M}\left(x\right),{\hat{p}}_{*}\left(x\right)\right)\right).$$
5. Calculate the increments (differences) of Jeffreys distance,
  (11) $$\begin{array}{c} \left(D_{1},\ldots ,D_{M-1}\right)=\left(\hat{J}\left({\hat{p}}_{2}\left(x\right),{\hat{p}}_{*}\left(x\right)\right)-\hat{J}\left({\hat{p}}_{1}\left(x\right),{\hat{p}}_{*}\left(x\right)\right),\;\;\ldots \;\;,\;\;\right.\\ \left.\hat{J}\left({\hat{p}}_{M}\left(x\right),{\hat{p}}_{*}\left(x\right)\right)-\hat{J}\left({\hat{p}}_{M-1}\left(x\right),{\hat{p}}_{*}\left(x\right)\right)\right). \end{array}$$
6. Use the LLR method to find the index $i^{*}$ in 1,…,M−1 corresponding to the change in scale of $\left(D_{1},\ldots ,D_{M-1}\right)$.
7. Consider the sub-samples $\left(D_{1},\ldots ,D_{i^{*}-1}\right)$ and $\left(D_{i^{*}},\ldots ,D_{M-1}\right)$. Use the LLR method to find the indexes $i^{**}$ and $i^{***}$ in $1,\ldots ,i^{*}-1$ and in $i^{*},\ldots ,M-1$, respectively, corresponding to changes in scale.

The procedure described above leads to detecting the indexes $i^{*}$, $i^{**}$, and $i^{***}$ which divide the increments of Jeffreys distance into four regimes. Interpretation of the determined results will be presented later in the paper, see Section 4, Section 5 and Section 6. Since the procedure is based on the pdfs in the subsequent segments, the method indicates the segment number, not the observation number, when the regime switches. However, in practice, we transfer that information into the number corresponding to the first observation in the determined segment, see Section 4 and Section 5. It is also important to mention that the procedure described in items 1–7 can be used twofold. Namely, while diving the signal into segments in item 1, one can consider the case of non-overlapping and overlapping windows. More precisely, for the non-overlapping case, as the first segment we take the samples with indexes from 1 to *L*, then we move by *L* samples and as the second segment we take the samples from 1+L to 2L and so on. The overlapping window corresponds to the case when as the first segment we take the samples from 1 to *L*, then we move by *l* samples and as the second segment we take the samples from 1+l to L+l and so on. As one can notice, when l=L we obtain the non-overlapping case. The choice of *l* affects the accuracy with which we indicate the moment of regime change. In Section 4 and Section 5 the value *l* is called step.

In items 6 and 7, to segment the increments of Jeffreys distance, we use the log-likelihood ratio method due to the fact that the scale parameter in $\left(D_{1},\ldots ,D_{M-1}\right)$ defined in Equation (11) changes in several regimes as the distribution of the signal varies with time, see the results presented Section 4 and Section 5. The LLR method enables detecting the moment of change in scale in the considered dataset by maximizing the log-likelihood ratio. For details of the method, we refer the readers, for example, to [87]. In the literature, there are known several methods designed for the same purpose (e.g., Regime Variance technique [22] and Absolute Median Deviation technique [17]), however, according to the simulations performed by the authors, the LLR method is the most powerful tool among those mentioned. The procedure described above is summarized in Algorithm 1. The most important points of the proposed procedure are the estimation of the pdf for data corresponding to the selected segments and calculation of the divergence measures (here Jeffreys distance). The last step is the application of the LLR method for the data representing the values of the divergence measure.
**Algorithm 1:** Segmentation procedure based on Jeffreys distance**Data**: Input data**Divide data into *M* segments of length equal to *L***: Segment 1, Segment 2, …, Segment M**Estimate the pdf in each segment**:
**_1_** **for**k←1**to***M***do****_2_** ⌊ Calculate ${\hat{p}}_{k}\left(x\right)$ for Segment k**Estimate the pdf of the last *L* samples in the signal**: ${\hat{p}}_{*}\left(x\right)$**Calculate the Jeffreys distance**:
**_3_** **for**k←1**to***M***do****_4_** ⌊ Calculate $\hat{J}\left({\hat{p}}_{k}\left(x\right),{\hat{p}}_{*}\left(x\right)\right)$**Calculate the increments of Jeffreys distance**:
**_5_** **for**k←1**to**M−1**do****_6_** ⌊ Calculate $D_{k}=\hat{J}\left({\hat{p}}_{k+1}\left(x\right),{\hat{p}}_{*}\left(x\right)\right)-\hat{J}\left({\hat{p}}_{k}\left(x\right),{\hat{p}}_{*}\left(x\right)\right)$**Use the LLR method to find change in scale in $\left(D_{1},\ldots ,D_{M-1}\right)$**: $i^{*}$Use the LLR method to find change in scale in $\left(D_{1},\ldots ,D_{i^{*}-1}\right)$: $i^{**}$Use the LLR method to find change in scale in $\left(D_{1},\ldots ,D_{i^{*}-1}\right)$: $i^{***}$

## 4. Results

**Figure 3:**
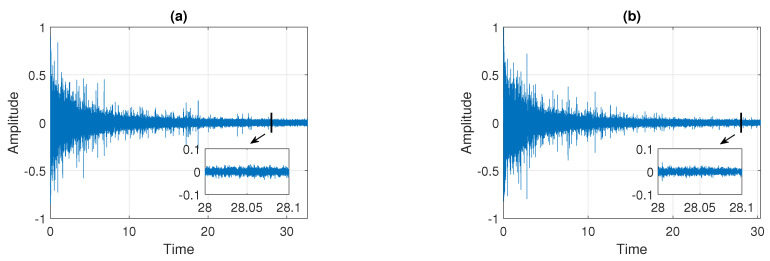
Examples of signals after the pre-processing step. Panel (**a**) corresponds to Signal 1 and panel (**b**) corresponds to Signal 2.

In this section, we present the results of applying the procedure described in Section 3.5 to the real signals presented in Figure 2. In the first step, the raw data are pre-processed by removing the deterministic components present in the signals. The pre-processing procedure is analogous to the one proposed in [30]. Examples of the cleaned signals, which are analyzed in detail in this section, are presented in Figure 3. Panel (a) corresponds to Signal 1 and panel (b) corresponds to Signal 2. One can see that they visually do not differ from the raw signals presented in Figure 2 (see panels (a) and (b)), however, the deterministic components present in the data are removed.

**Figure 4:**
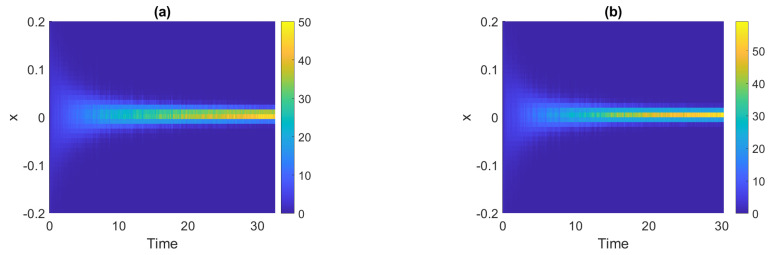
Density maps for Signal 1 (panel (**a**)) and Signal 2 (panel (**b**)).

To illustrate how the distribution changes over time, for the signals presented in Figure 3 we estimate the pdfs in non-overlapping windows of length 2500. The obtained density maps are shown in Figure 4, where panel (a) corresponds to Signal 1 and panel (b) corresponds to Signal 2. One can see that the values tend to be more concentrated around zero over time, i.e., the functions take extreme values with smaller probability. It confirms that the dispersion statistics are smaller as time goes. Moreover, we can notice that the pdfs at the end of the signals, at least visually, are very similar (or almost identical), i.e., the distribution stops changing after a certain point.

**Figure 5:**
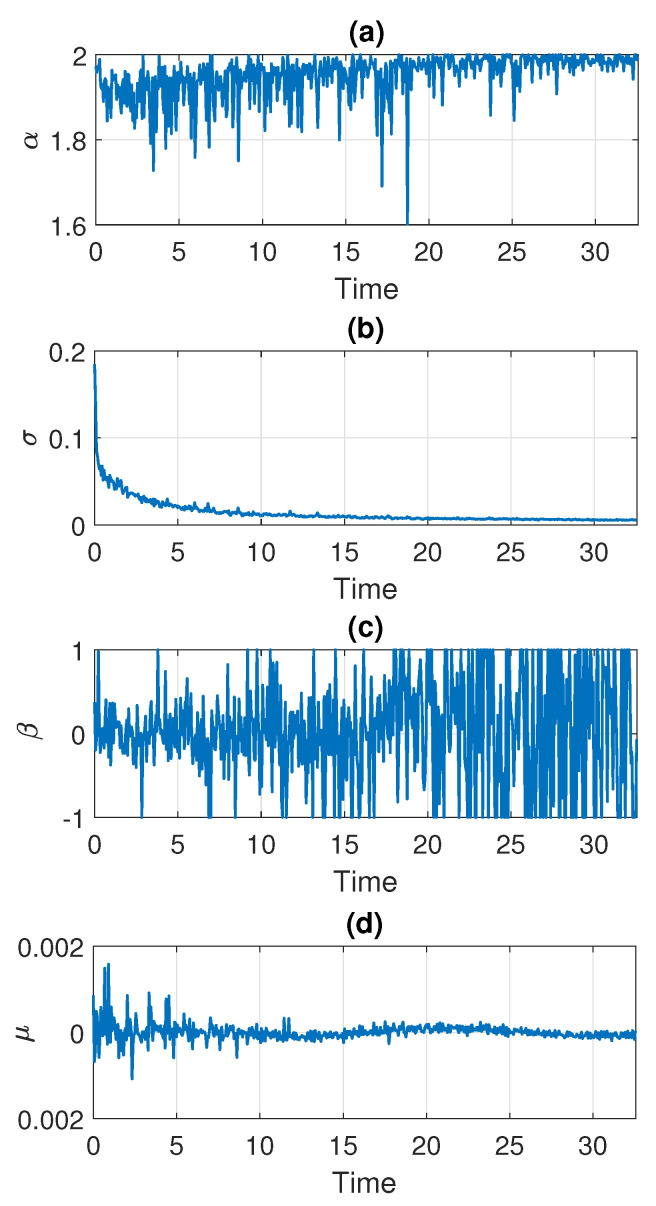
Estimated parameters of the stable distribution for Signal 1.

**Figure 6:**
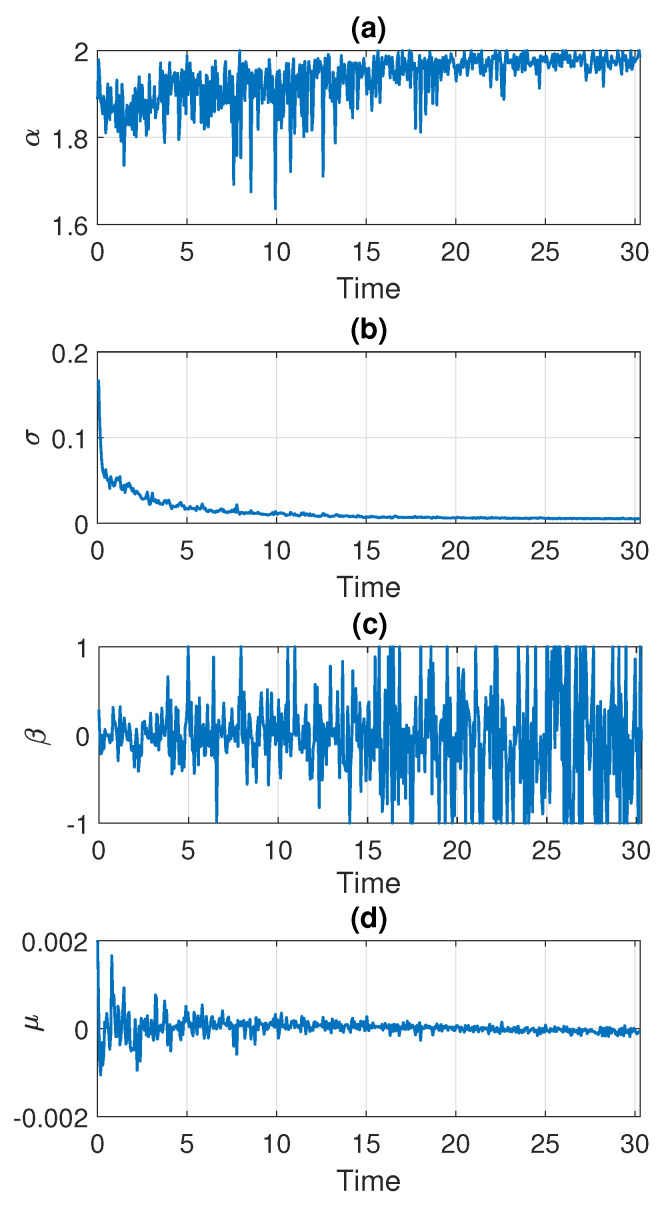
Estimated parameters of the stable distribution for Signal 2.

In the following part, we estimate the parameters of the stable distribution, described in Section 3, in non-overlapping windows of length 2500. The results for Signal 1 are presented in Figure 5 and for Signal 2 in Figure 6. Panels (a), (b), (c), and (d) correspond to α, σ, β, and μ, respectively. One can notice that the calculated values change over time. For both examples, the stability index α takes values between 1.6 and 2. Moreover, one can see an upward trend with time and at the end of the signal the values stabilize very close to 2 which means that the distribution is similar to the Gaussian one. Since the parameter α in panel (a) is related to the probability of occurrence of impulses, such behavior is natural as the number of impulses in the signal decays with time. This is consistent with our assumption that the distribution of the data smoothly transforms and stabilizes with time. The values of skewness parameters presented in panel (c) also indicate that the distribution is getting close to Gaussian with time since at some point β begins to take values from the entire interval [−1,1]. This is related to the fact that the skewness parameter becomes irrelevant for the Gaussian distribution (see Equation (2) for α=2). The values of the scale parameter σ presented in panel (b) also decrease with time, which agrees with the results seen on the density maps. One can see the exponential-type decaying, so we can assume that from a certain point σ stabilizes to a certain level. The location parameter μ presented in panel (d) is always close to zero.

**Figure 7:**
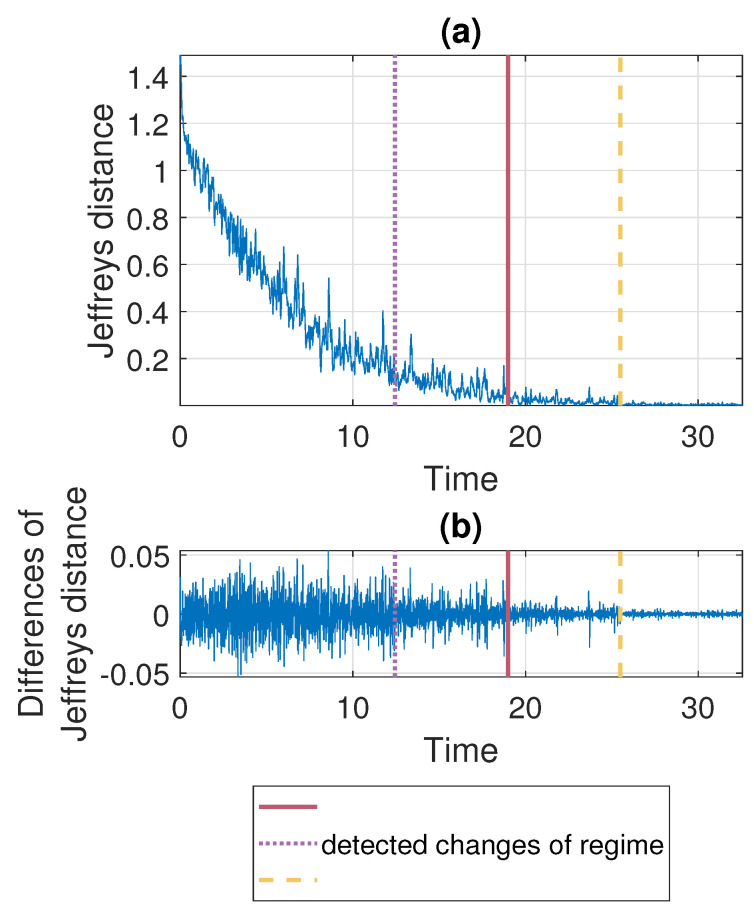
Jeffreys distance (panel (**a**)) and differences of Jeffreys distance (panel (**b**)) comparing the pdfs in the moving window of length 2500 with step equal to 250 to the pdf corresponding to the last window for Signal 1. Detected regime change points are marked in purple (dotted line), red (solid line), and yellow (dashed line).

**Figure 8:**
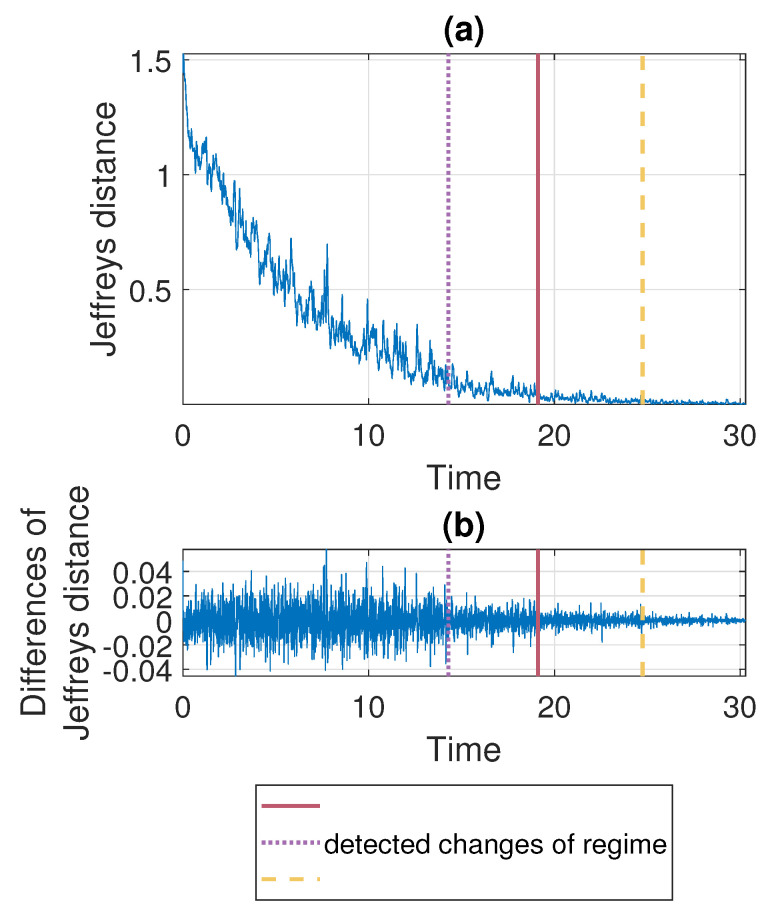
Jeffreys distance (panel (**a**)) and differences of Jeffreys distance (panel (**b**)) comparing the pdfs in the moving window of length 2500 with step equal to 250 to the the pdf corresponding to the last window for Signal 2. Detected regime change points are marked in purple (dotted line), red (solid line) and yellow (dashed line).

**Figure 9:**
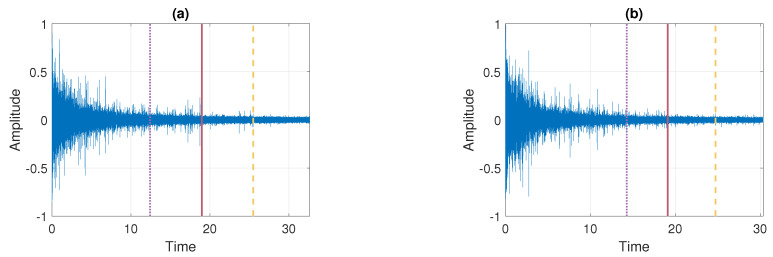
Signals with marked regime changes. The first change is visible as a dotted purple line, second: a solid red line, and the last one is a dashed yellow line. Panel (**a**) corresponds to Signal 1 and panel (**b**) corresponds to Signal 2.

According to the segmentation procedure presented in Section 3.5, now we compare the pdfs of the signal in the moving windows of length 2500 with the pdf estimated based on the last 2500 samples. The comparison is done using the Jeffreys distance. For Signal 1 and Signal 2 the values of the measure and its differences are presented in Figure 7 and Figure 8, respectively. One can notice that the values of Jeffreys statistics decrease and therefore we can conclude that the distributions become more similar to the distribution in the last window or, in other words, they stabilize. We recall here that the Jeffreys distance equal to 0 indicates that the pdfs are the same. Moreover, the behavior of the statistics increments also changes with time, i.e., the values taken by Jeffreys distance get more stabilized as time goes (there are fewer oscillations), which is visible in panel (b), showing the differences in the statistics. From about 25 s on, the values of Jeffreys distance are very close to 0, and at the same time, their differences are small. Using the above observations, we propose a method described in Section 3 which relies on dividing the values of Jeffreys distance into four separate regimes with a constant scale parameter of their differences. According to this, the point marked with the solid red line was detected first. Then the values preceding and following this point are also divided into two regimes using the same method. This leads to the designation of four regimes. For the signals presented in this section, the regimes are as follows. For Signal 1, the first regime change occurs in 12.437 s (denoted by the dotted purple line), the second regime change occurs in 18.985 s (solid red line), and the third regime change occurs in 25.4889 s (dashed yellow line). For Signal 2 we have: 14.291 s, 19.1156 s, and 24.7449 s. One can observe that the behavior of Jeffreys distance changes in the detected segments. Namely, in the last regime (after the dashed yellow line) the values are very close to 0 (the pdfs are almost identical) and there are few oscillations. In the second last regime (between solid red and dashed yellow lines) the pdfs are also very similar, however, we observe more oscillations of the values in comparison to the last regime. This different behavior of the Jeffreys distances in the third and fourth regimes may be caused by the impulses that still occur in the signal related to the third regime more frequently despite the fact that the dispersion of the data is similar in both intervals. The division into the first and the second regimes (separated by the dotted purple line) is mainly related to the change in the rate of decline of the values taken by the Jeffreys distance. We mention here that the proposed procedure leads to the indication of a specific window in which the behavior of the values taken by the Jeffreys distance changes. Here, as the results, we present a moment (in seconds) corresponding to the number of the first observation in the window indicated by the procedure. Since we compare the pdfs in moving windows, the accuracy of the method is related to the step in which we move windows. Therefore, we consider three cases here: a moving overlapping window of length 2500 with step equal to 250, a moving overlapping window of length 2500 with step equal to 500, and a moving non-overlapping window of length 2500 (equivalent to step equal to 2500). The results presented in Figure 7 and Figure 8 correspond to the step equal to 250. However, Table 1 contains the results for all signals with three different steps (250,500,2500). For the considered signals, the detected regime change points, which are shown in Figure 7 and Figure 8, are also plotted on the cleaned signals, see Figure 9.

As mentioned, the procedure is repeated for all signals presented in Figure 2 while considering different values of steps. The results obtained for the chosen step values (see Table 1) are usually similar, although in a few cases there are larger discrepancies (purple point for Signal 2, Signal 3 and Signal 7 for step 2500; red point for Signal 3 for step 2500, and yellow point for Signal 7 for step 2500). Most often, these discrepancies appear when detecting the purple point, see also the results for the simulated signals presented in Section 5. One can notice that for different signals, the obtained results of change regime points are not the same. It is intuitive, because for each experiment there is a different arrangement of the grains in the grinding mill, which affects the grinding speed, and thus affects the data distribution over time. The first regime change point (purple) occurs at the earliest for Signal 5 (around 9 s) and the latest for Signal 6 (around 15 s) or for Signal 3 (where only the non-overlapping window method indicates a value close to 16 s). The second regime change point (red) occurs at the earliest for Signal 5 (around 16 s) and the latest for Signal 7 (over 21 s). The last point (yellow) is determined at the earliest for Signal 8 (about 21 s), and at the latest for Signal 1 and Signal 4 (between 25 s and 26 s). For Signal 7 and the step equal to 2500 we obtain a clear outlier (the procedure indicates 29 s).

## 5. Simulations

In this section, we apply the introduced methodology to the simulated signals constructed based on the assumptions mentioned in Section 3.2 and Section 3.3, i.e., for a sequence of independent random variables from the symmetric stable distribution with stability index and scale parameter changing over time. As it was mentioned before, for the real signals considered in Section 4 we do not know the exact moments when the characteristics of the process stabilize. Here, for the simulation study purpose, we set the moments at which the parameters α and σ remain unchanged, and therefore we are able to validate the efficiency of the proposed procedure.

**Figure 10:**
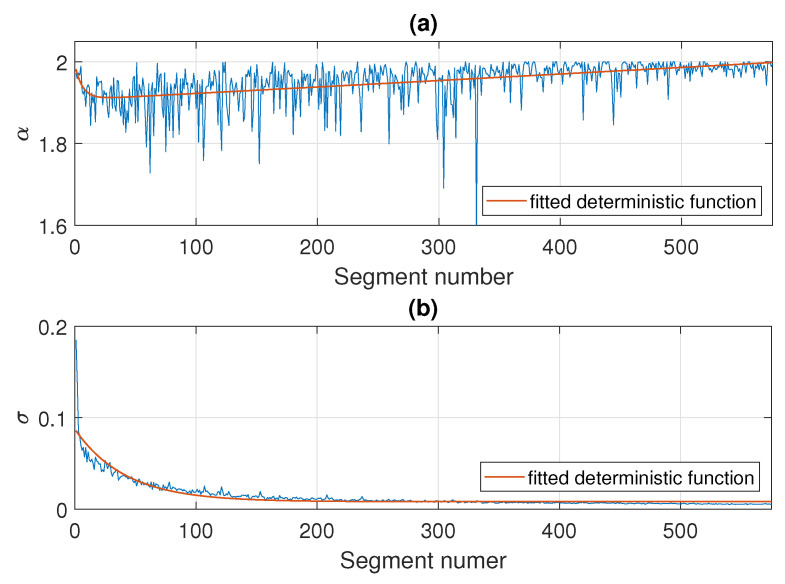
Estimated values of α on panel (**a**) and σ on panel (**b**) in the subsequent segments of length 2500 for Signal 1. Fitted deterministic functions are marked in red.

As an illustration, in Figure 10 we present the estimated values of α and σ for Signal 1 examined in detail in Section 4. The parameters are calculated in non-overlapping windows of length 2500. Here, the number of segments is equal to 575 and, according to the results presented in the previous section, the regimes change around the segment of number 200, of number 350, and of number 450. Under the taken assumptions, the stability and scale parameters change with some deterministic trends, namely, a sum of two exponential functions for α and a exponential function for σ, respectively, which are marked in red in Figure 10.

In the following part of this section, we examine the simulated signals consisting of, similarly to the real signal mentioned above, 575 segments with 2500 independent symmetric stable random variables in each of them. The parameters of the distribution in the subsequent segments change according to the deterministic functions fitted to the parameters α and σ for Signal 1 (Figure 10) disturbed by the zero-mean Gaussian noise with standard variation equal to 0.03 for the stability index and 0.0005 for the scale parameter. Additionally, as it was mentioned before, we fix the values of α and σ at a certain point. The moments of σ stabilization and α stabilization are chosen to correspond to the change of regimes detected for Signal 1 in the previous section, i.e., the scale parameter and the stability index remain unchanged from the segment of number 351 and of number 451, respectively.

**Figure 11:**
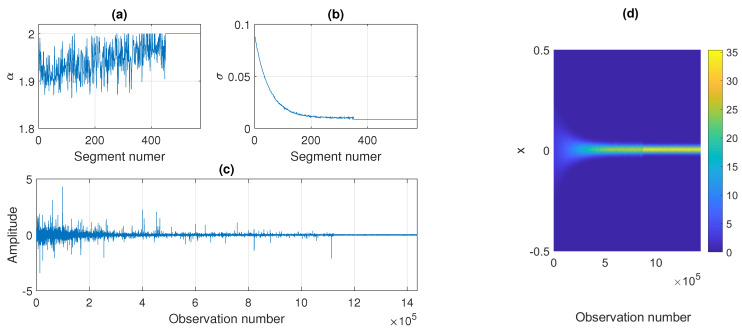
Values of α (panel (**a**)) and σ (panel (**b**)) in the subsequent segments of length 2500, the obtained simulated signal (panel (**c**)), and the corresponding probability density map (panel (**d**)).

The construction of the sample simulated signal is presented in Figure 11. Panels (a) and (b) show the theoretical values of α and σ in the subsequent segments, whereas panel (c) presents the obtained trajectory. In panel (d) we also show the corresponding map of the pdfs calculated in the subsequent non-overlapping segments of length 2500. As one can see, the map looks similar to the ones presented for the real signals in Figure 4. The pdfs become more concentrated around zero with time: the functions take extreme values with smaller probabilities. It should be emphasized that in the plot given in panel (d) of Figure 11 there is a noticeable change in the pdfs after 350 segments (around 875,000 sample) which results from the stabilization of the scale parameter. However, the stabilization of the stability index after 450 segments (about 1,125,000 sample) is barely visible in the pdf map. Nevertheless, by using the proposed procedure, one can also detect this change.

**Figure 12:**
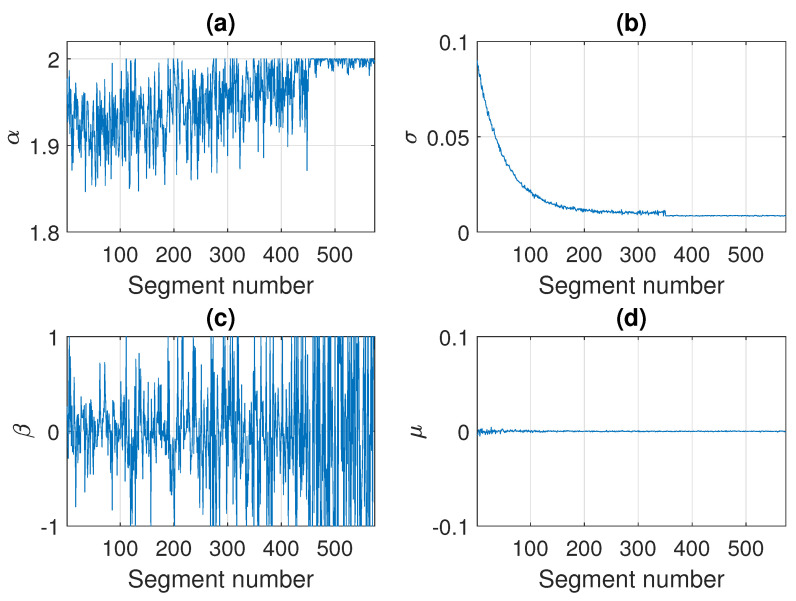
Estimated parameters of the stable distribution for sample simulated signal presented in panel (**c**) of Figure 11. Panels (**a**), (**b**), (**c**) and (**d**) correspond to α, σ, β and μ, respectively.

In Figure 12 we present the parameters of the stable distribution estimated in non-overlapping windows of length 2500 for the trajectory presented in Figure 11. The estimated values of α and σ are close to the theoretical ones. It is worth noticing that for the stability index of the segment of number 451 the estimated values are not equal to exactly 2, which is the value set while simulating, but they are close to 2 with some oscillations. Since the generated observations are symmetric stable, the shift parameter μ is close to 0 for all segments, and the skewness parameter β from a certain point takes values in the whole interval [−1, 1], because the distribution is very close to Gaussian (or even Gaussian after 450 segments).

**Figure 13:**
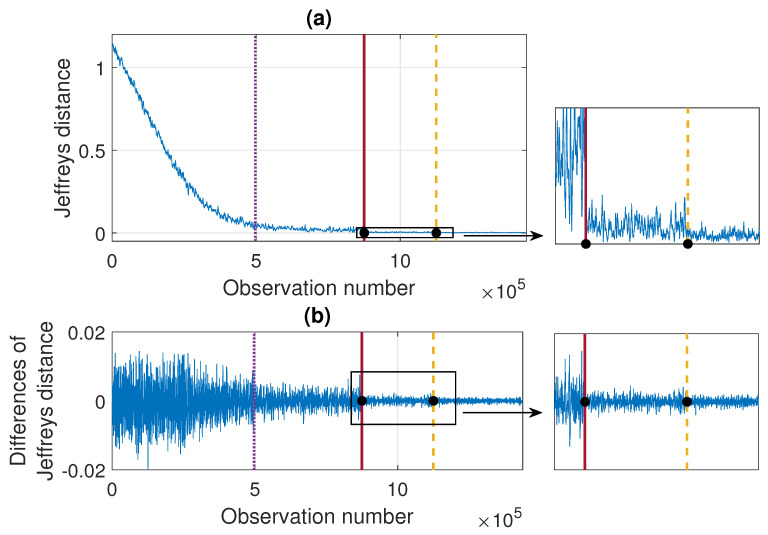
Jeffreys distance (panel (**a**)) and differences of Jeffreys distance (panel (**b**)) comparing the pdfs in the moving window of length 2500 with step equal to 250 to the the pdf corresponding to the last window. Detected regime change points are marked in purple (dotted line), red (solid line), and yellow (dashed line) and the theoretical moments of σ and α stabilization are marked with black dots.

**Figure 14:**
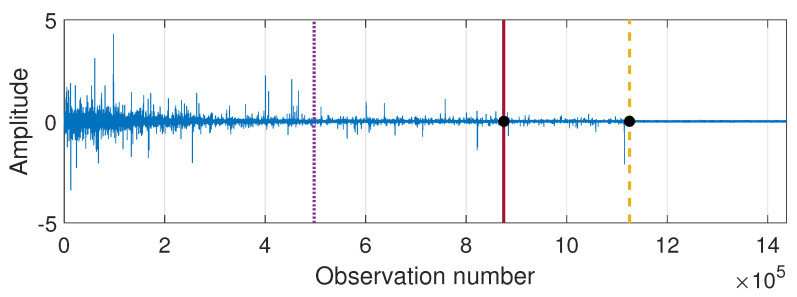
Simulated signal with regime change points marked in purple (dotted line), red (solid line), and yellow (dashed line) and the theoretical moments of σ and α stabilization marked with black dots.

In Figure 13 we present the results of applying the proposed procedure to the considered sample simulated signal. Panel (a) shows the values of the Jeffreys distance and panel (b) presents the differences of Jeffreys distance on the basis of which the regime change points are determined and marked, respectively, in purple (dotted line), red (solid line), and yellow (dashed line). Additionally, the theoretical regime change points (corresponding to stabilization of σ and α) are marked with black dots. One can see that the values of Jeffreys divergence decrease with time and at the same time the rate of decline of the function changes. The last two regimes, detected using the proposed method, are related to the stabilization of the scale parameter and the stability index, respectively. The determined moments of regime changes in σ and in α coincide with the theoretical ones (red solid line and yellow dashed line, respectively). Finally, Figure 14 shows the trajectory of the simulated signal with the theoretical and detected regime change points marked with lines and dots, respectively. We mention here that the results presented in Figure 13 are obtained by comparing the successive pdfs determined in the overlapping windows of length 2500 with the pdf in the last window. The windows overlap since we shift by 250 observations when counting the successive densities.

**Figure 15:**
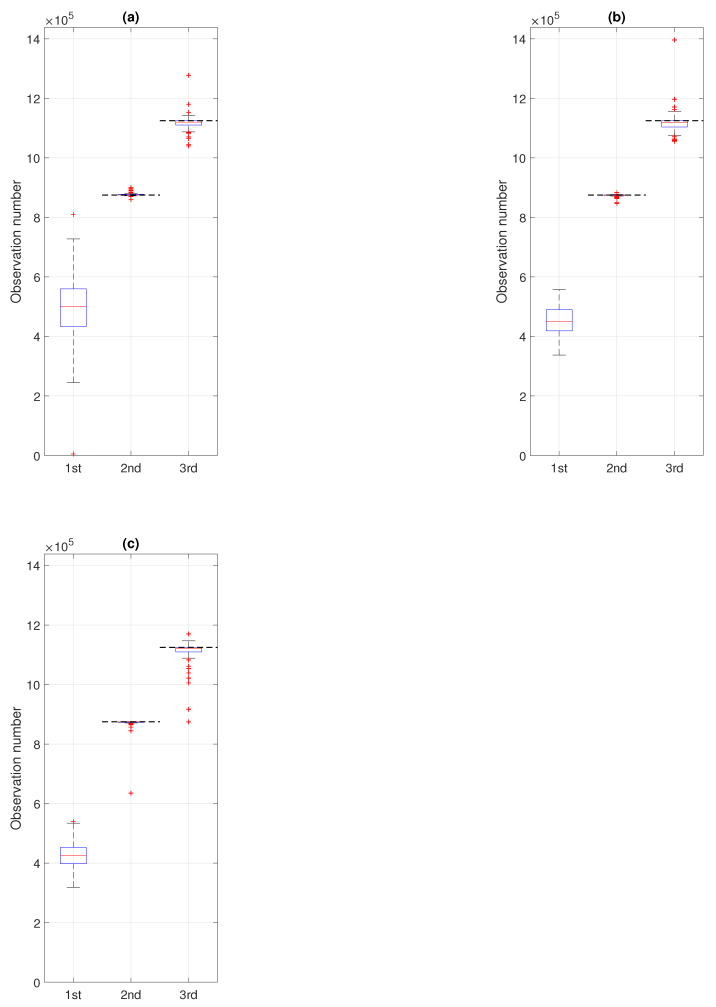
Boxplots presenting the results of the Monte Carlo simulation study, i.e., the identified moments of the first (purple), the second (red), and the third (yellow) regime changes for 100 simulated signals. Panel (**a**) corresponds to the procedure applied to non-overlapping windows of length 2500 (with step equal to 2500) and panels (**b**) and (**c**) correspond to the overlapping windows of length 2500 with step equal to 500 and 250, respectively. Black dash lines for the second and the third boxplot indicate the theoretical moments of σ and α stabilization, respectively.

In the last step, we verify the efficiency of the proposed methodology by conducting a Monte Carlo simulation study for a number of signals generated analogously to the one with the trajectory presented in panel (c) of Figure 11. Namely, we simulate 100 signals (each one with different values of σ and α in the subsequent segments, see panels (a) and (b) of Figure 11) and to each of them, we apply the proposed procedure. As a result, we obtain 100 values corresponding to the moments of regime change: the 1st change (purple), the 2nd change (red), and the 3rd change (yellow). The outcomes are presented in the boxplots given in Figure 15, where panel (a) corresponds to the case of comparing the pdfs in non-overlapping windows of length 2500 (with step equal to 2500) and panels (b) and (c) correspond to the case of comparing the pdfs in overlapping windows of length 2500 (with step equal to 500 or 250, respectively). For the second and third regime changes, the theoretical values (i.e., the observation numbers) related to the stabilization of the scale parameter (observation of number 875,000, i.e., after 350 segments of length 2500) and of the stability index (observation of number 1,125,000, i.e., after 450 segments of length 2500) are marked with horizontal black dashed lines. We remind here that our procedure leads to detecting the window number, however, we transfer that information to the number of the first observation in the identified window. As one can see in Figure 15 for the second and the third regime change, the estimated values are close to the theoretical moments of σ and α stabilization. That can also be seen in Table 2 where we present the median, interquartile ranges, and 80% quantile intervals calculated based on the results of the Monte Carlo study. For the first regime change, for which we do not have the theoretical equivalent, we can see that the medians, IQRs, and the length of quantile intervals get smaller as the step decreases, whereas for the second and third regime change the medians are similar for all three values of the step and they are close to the theoretical moment of stabilization. Moreover, the IQRs and the length of quantile intervals are smaller for the second regime change than for the third one, which indicates that the moment of second regime change (related to the scale parameter) is detected with higher precision, which can also be seen in Figure 15.

## 6. Discussion and Validation of the Procedure

**Figure 16:**
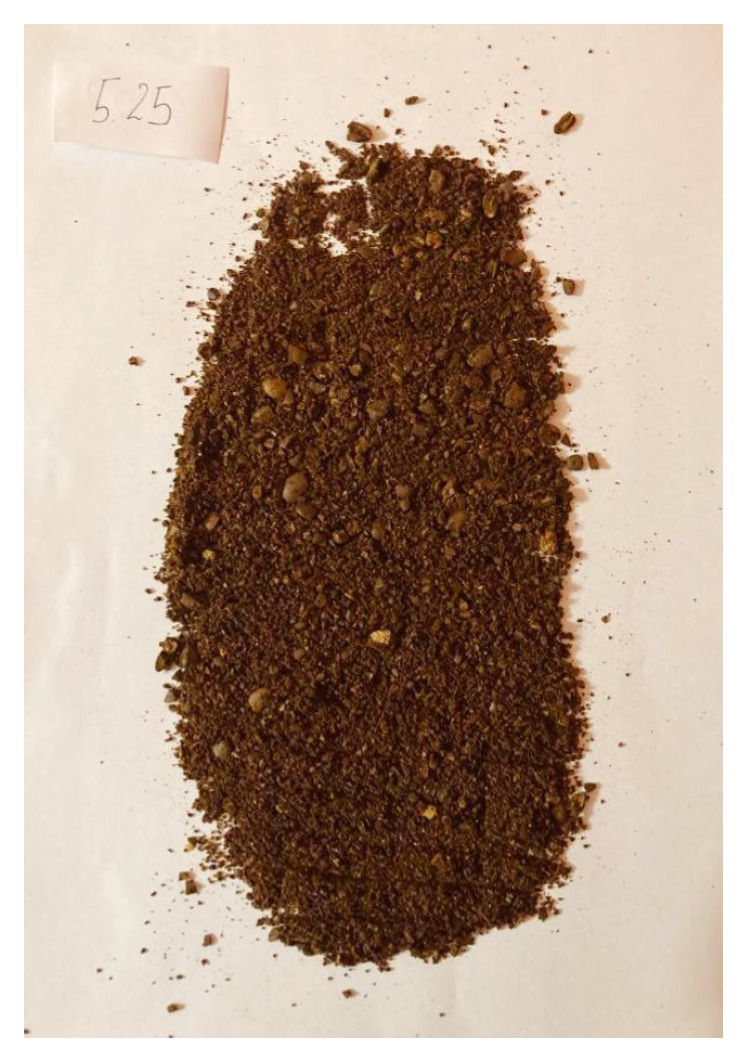
Coffee beans ground for about 5 s.

**Figure 17:**
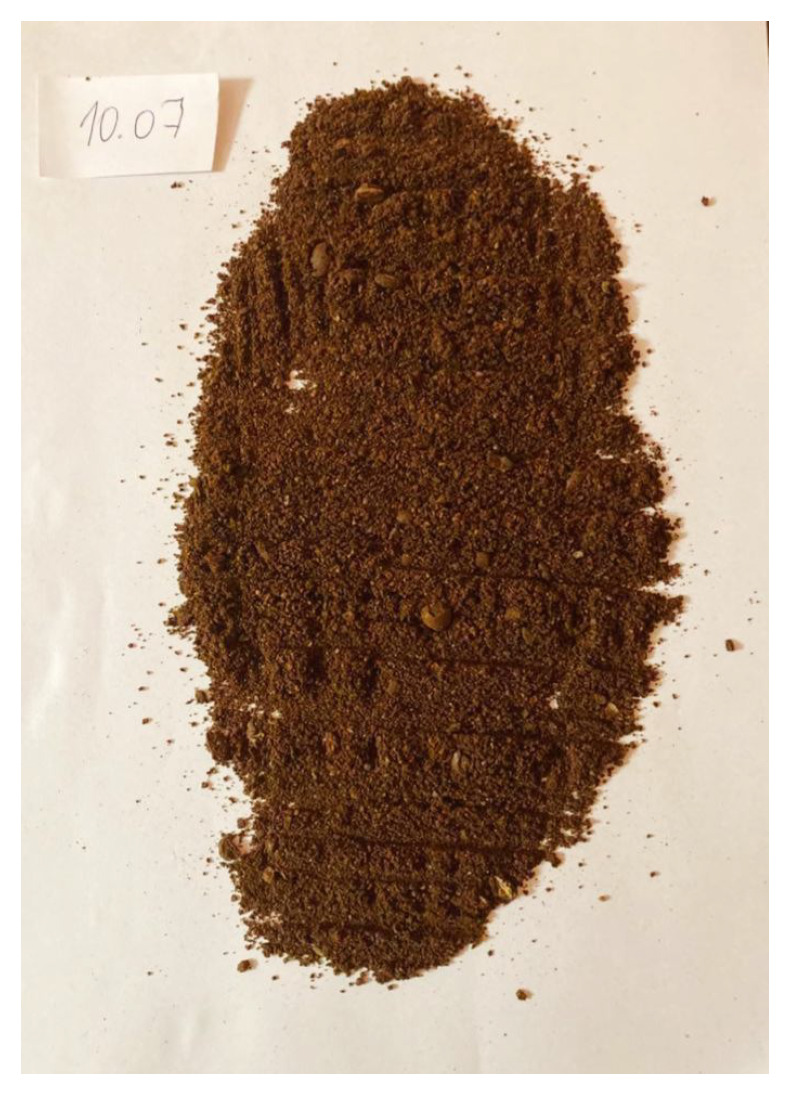
Coffee beans ground for about 10 s.

**Figure 18:**
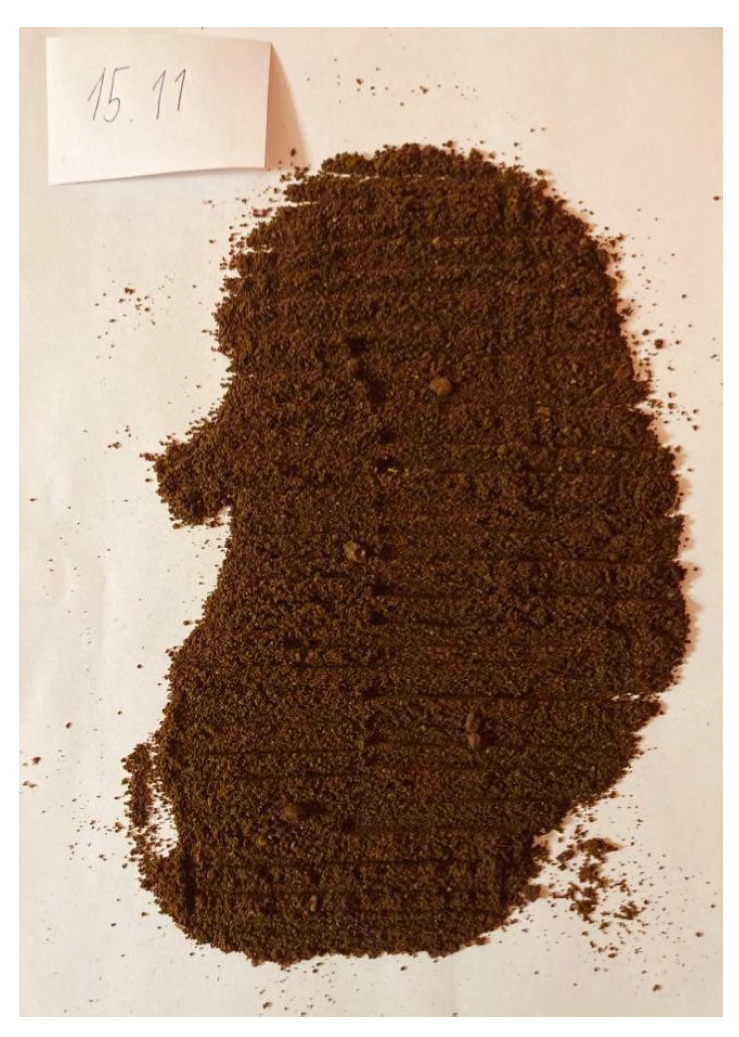
Coffee beans ground for about 15 s.

**Figure 19:**
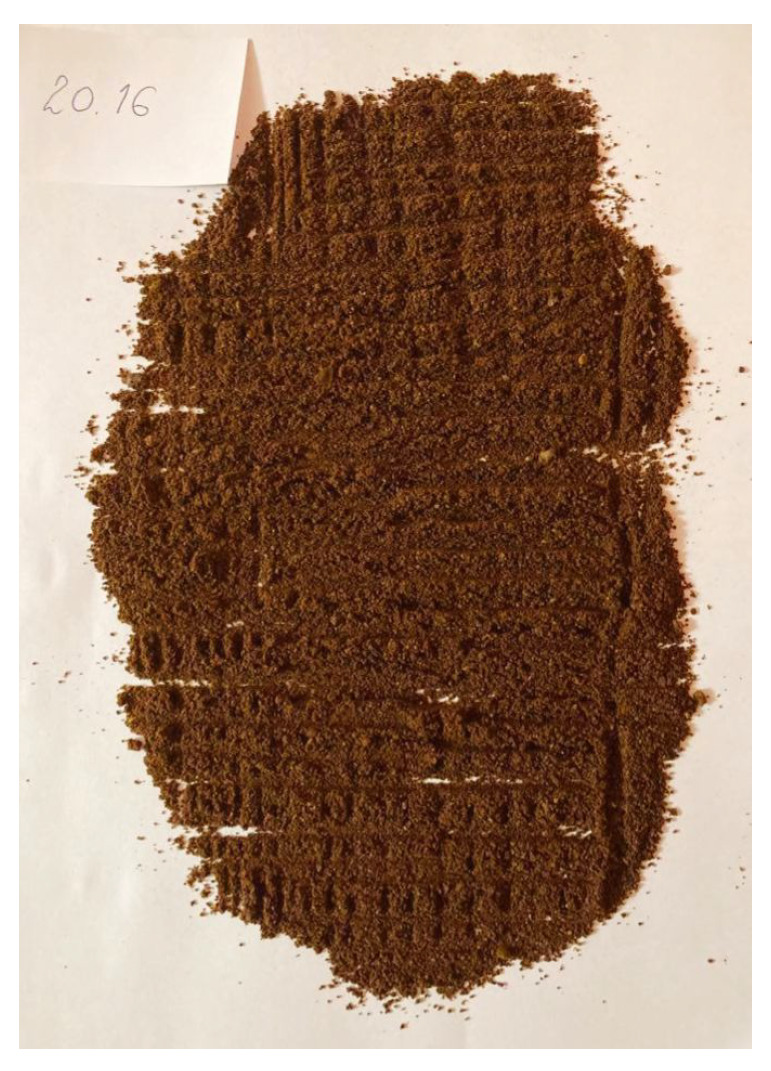
Coffee beans ground for about 20 s.

**Figure 20:**
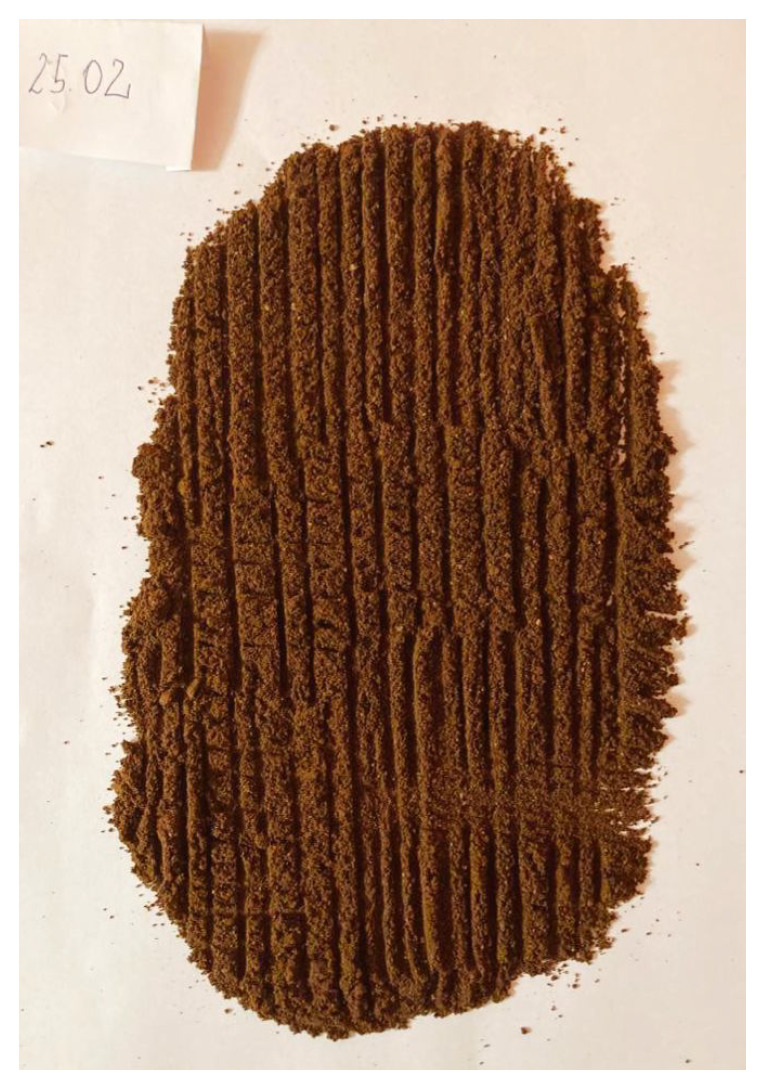
Coffee beans ground for about 25 s.

**Figure 21:**
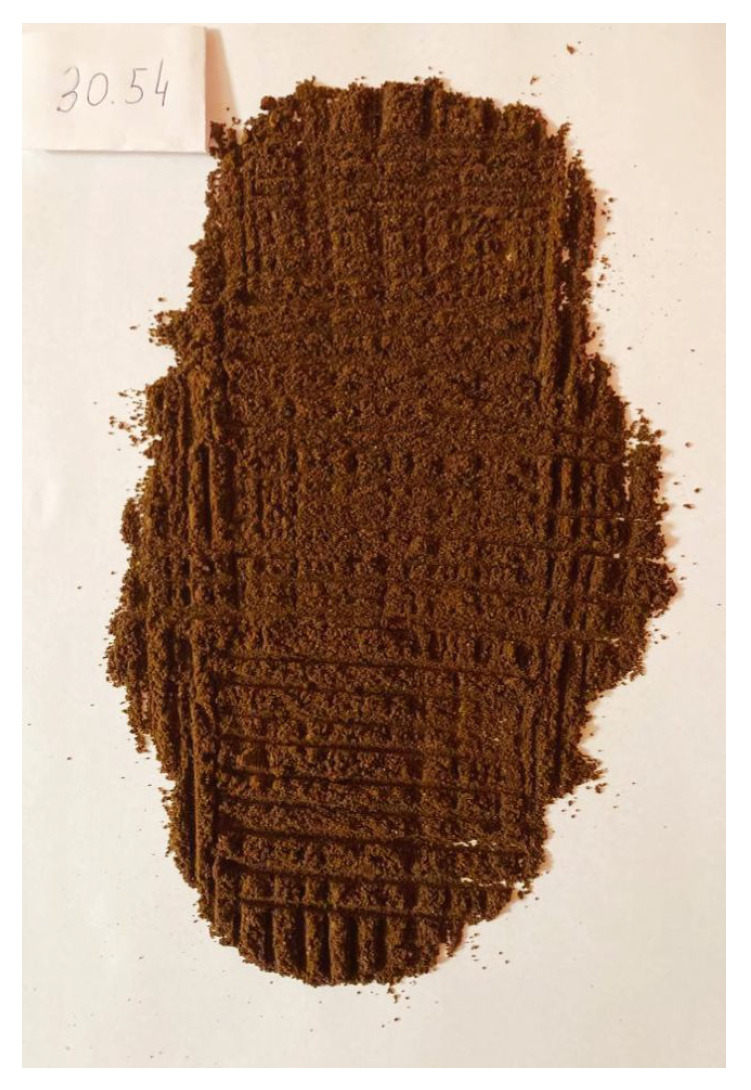
Coffee beans ground for about 30 s.

To validate the obtained results, we repeated the experiment of coffee grinding several times. With each repetition, we extended the grinding time and took photos of the ground coffee beans after the experiment was completed. The same amount of coffee was ground for about 5 s, 10 s, 15 s, 20 s, 25 s, and 30 s sequentially. Pictures of the product obtained after grinding are shown in Figure 16, Figure 17, Figure 18, Figure 19, Figure 20 and Figure 21, respectively. One can notice that the structure of the coffee beans is clearly grainy in the photos presented in Figure 16 and Figure 17, i.e., after 5 s and 10 s of grinding. Then, as expected, the longer the coffee beans are ground, the more powdered the product becomes. Nevertheless, in the picture shown in Figure 18, i.e., after 15 s, and even in the picture presented in Figure 19, i.e., after 20 s, one can see individual unground or half-ground coffee beans. In the last two photos in Figure 20 and Figure 21, corresponding to a grinding time of 25 s and 30 s, the appearance of the ground product is very similar. The structure is powdered without individual coffee beans in the product. This is in line with the results presented in Section 4 for real signals, which indicate that after about 24 s, on average, the probability distribution in the acoustic real signals stabilizes (the arithmetic mean of the values in the last column in Table 1 is equal to 24.0371 s).

The preliminary evaluation of the results based on the photos is also supported by the analytical results. We remind here that the method proposed in Section 3.5, leading to the segmentation of data, is designed in such a way that the signal is first split into two regimes, and then each regime is again split into two segments. As a result, we obtain the signal segmented into four regimes within which the pdfs of the data show certain similarities. To confirm the interpretation based on real data analysis, a simulation study was carried out. The outcomes presented for the simulated signals confirmed our assumption that the regime change related to data dispersion is detected first (marked in red on the plots). Detecting this regime change initially splits the data into two parts and in the second one, the scale parameter is stabilized. Then, as mentioned above, each of the two segments is divided once again into two regimes. For the part of the signal corresponding to the regime with stabilized dispersion, this step detects the moment when the impulsivity in the data stabilizes (marked in yellow on the plots), i.e., it distinguishes between non-Gaussian and Gaussian data. For the part of the signal corresponding to the regime with non-stabilized dispersion, the above step also leads to detecting the regimes with different behavior of the pdfs (marked in purple on the plots), but it does not have an interpretation related to any parameter.

As mentioned in Section 2, the problem formulated in the paper concerns data segmentation where the parameters of the probability distribution change over time. These considerations were motivated by real data, in which the changes in the probability distribution are not sudden but occur smoothly and at some point, the parameters stabilize at a certain level. In the analyzed signals, both real and simulated, the scale and impulsivity of the data changed over time. To segment the signals, we proposed a method based on the assessment of the similarity of the pdfs in the moving windows. The proposed procedure allows determining the moments at which the scale parameter and the parameter responsible for the impulsivity of the data stabilize. For the analyzed real signals, the last detected moment of regime change, indicating that the segment with stabilized amplitude and stabilized impulsivity in the data, can answer the question of how long coffee should be ground to achieve a satisfactory effect. Since the probability distribution of the acoustic signal does not change from that moment on, we can conclude that the structure of the ground product also remains unchanged and the ground coffee obtains its final structure.

## 7. Conclusions

In this paper, an original signal segmentation procedure for a random process with time-varying characteristics is proposed. Typically, the signal segmentation process is related to the detection of a moment in time when the process A switches to process B. The situation is relatively simple when the segments contain data described by different distributions or the corresponding distribution is the same but the parameters are different in those segments. The rule is simple here: the bigger the difference between the segments, the easier the segmentation. In that case, the segmentation algorithm divides the data into homogeneous parts.

The case considered in this paper is much more complicated. The analyzed input data correspond to the model given by Equation (1). In that case, the distribution of the time series is the same, however, we assume that the parameters are time-varying deterministic functions which can be even disturbed by random noise. The examined process is a specific one. It is highly non-stationary, strongly non-Gaussian (impulsive) at the beginning, during the single realization, transforming to a nearly Gaussian process with much smaller amplitudes.

Stable distribution with varying parameters has been proposed to describe the data. For some combination of values of its parameters (α, σ) the signal could be impulsive or not, may have higher “energy”, or may be weak. It is perfectly matched with the analyzed case. However, as it was mentioned, the segmentation algorithm does not use the assumption of stable distribution of the data. It could be generalized to any signal containing two processes with transitions or simply switched from A to B.

To identify the difference between parts of the signal, we used segmentation with a priori predefined length of the segment with two versions: without or with overlapping. For each segment, the probability density function is estimated and the difference between them is evaluated by distance measurement (Jeffreys distance). The final step is to find (using LogLikelihood ratio LLR) the change point in the differentiated distance time series. Note that there is just one important parameter (segment size). For longer segments, we will improve the quality of pdf estimation, but we will lose the resolution in time. To minimize this effect, overlapping is used. The method is pretty intuitive and universal.

To illustrate the problem and provide evidence of the effectiveness of the proposed method, two approaches have been proposed. A model of the signal has been proposed using the mentioned α-stable distribution with parameters changing in time. We applied the Monte Carlo approach to validate the segmentation efficiency statistically. Obtained results were very good. Next, an acoustic signal acquired during the grinding of coffee beans in a grinder has been used. It was found that the process of grinding of coffee beans matches our research problem perfectly. In the beginning, due to the cutting of beans by sharp spinning knives in the grinder, the process is very noisy. Moreover, the cutting process is an impulsive one. Once the coffee beans are ground, the material in the grinder is more powdery than grainy. The acoustic signal is much more narrow-band (no impulses) due to rotating elements. The transition between processes is smooth—thus, the segmentation is complicated and the regime change point is much more difficult to detect. To prove the quality of the results for the real data, we prepared the photo documentation of the experiment. Based on the proposed method, the identified change point corresponds to low granularity of the coffee.

Both approaches (photos and simulations) provide similar information, so we assume that the method is appropriate and effective.

We believe that the problem discussed in the paper may be important in many engineering applications (for example, impulsive noise/vibration with damping). One may assume also that other specific parameters of the process can vary simultaneously. Therefore, further work might be related to validation on other real cases as well as generalization of segmentation to any processes with smooth transition.

## Figures and Tables

**Table 1:** Detected regime change points (in seconds) for all eight signals for different values of step at which the window of length 2500 is moved.

Signal Number	Step	Purple Point	Red Point	Yellow Point
1	2500	11.8481	18.9909	25.5669
	500	12.4376	18.7755	25.4989
	250	12.437	18.9850	25.4889
2	2500	12.6984	19.1043	24.7732
	500	14.2857	19.1156	24.7392
	250	14.2914	19.1156	24.7449
3	2500	15.8163	18.7642	22.1088
	500	11.0544	16.9841	22.0295
	250	11.0658	17.0465	21.9728
4	2500	12.8685	19.7279	25.6236
	500	12.8458	18.2880	25.5556
	250	12.8231	18.4751	25.5612
5	2500	9.3537	15.7029	21.9388
	500	9.3651	15.9864	21.9728
	250	9.3594	16.0431	22.0125
6	2500	15.0794	18.7642	24.8299
	500	15.1020	18.7642	24.5896
	250	15.0794	18.7642	25.7959
7	2500	10.9410	21.5986	29.3084
	500	14.6825	21.3605	24.6145
	250	14.6825	21.3719	25.1304
8	2500	11.2245	15.8163	21.3152
	500	12.3243	15.8050	20.8617
	250	12.3186	15.7937	20.8560

**Table 2:** Medians, intequartile ranges (IQR), and 80% quantile intervals calculated based on the identified moments of the first (purple), the second (red), and the third (yellow) regime changes for M=100 simulated signals for the procedure applied to non-overlapping windows of length 2500 with step equal to 2500 and to the overlapping windows of length 2500 with step equal to 500 and 250.

	Purple Point	Red Point	Yellow Point
theoretical value	-	875,000	1,125,000
step = 2500			
$Q_{0.50}$	500,000	875,000	1,120,000
IQR	126,250	2500	15,000
$\left[Q_{0.10};Q_{0.90}\right]$	[395,000; 610,000]	[872,500; 883,750]	[1,093,750; 1,131,250]
step = 500			
$Q_{0.50}$	450,000	874,500	1,118,500
IQR	71,250	1500	21,250
$\left[Q_{0.10};Q_{0.90}\right]$	[390,250; 523,250]	[870,750; 875,500]	[1,079,250;1,140,250]
step = 250			
$Q_{0.50}$	425,750	874,250	1,120,750
IQR	54,750	2000	15,625
$\left[Q_{0.10};Q_{0.90}\right]$	[378,125; 478,000]	[869,000; 875,000]	[1,090,125; 1,128,625]

## Data Availability

Data available on request.
